# Impact of high intensity interval and moderate continuous training on plasma ratios of ProBNP_1-108_/BNP_1-32_ and NT-pro-BNP_1-76_/BNP_1-32_ after coronary artery bypass grafting surgery

**DOI:** 10.3389/fphys.2023.1114813

**Published:** 2023-03-07

**Authors:** Sara Zare Karizak, Majid Kashef, Abbas Ali Gaeini, Mostafa Nejatian

**Affiliations:** ^1^ Sport Science Department, Faculty of Literature and Humanities, Persian Gulf University, Boushehr, Iran; ^2^ Exercise Physiology Department, Sport Science Faculty, Shahid Rajaee Teacher Training University, Tehran, Iran; ^3^ Exercise Physiology Department, Sport Science Faculty, Tehran University, Tehran, Iran; ^4^ Cardiac Rehabilitation Specialist and Head of Rehabilitation Clinic of Tehran Heart Center Hospital, Tehran, Iran

**Keywords:** high intensity interval training (HIIT), moderate continuous training (MCT), ProBNP_1-108_/BNP_1-32_, NT-pro-BNP_1-76_/BNP_1-32_, CABG surgery

## Abstract

**Background:** ProBNP_1-108_/BNP_1-32_, and NT-pro-BNP_1-76_/BNP_1-32_ ratios are significant indices for predicting complications after coronary artery bypass grafting (CABG) surgery. However, the effect of aerobic training types on these biomarkers has not been fully understood. So, the current study aimed to determine the impact of aerobic interval and continuous training programs on plasma ratios of ProBNP_1-108_/BNP_1-32_ and NT-pro-BNP_1-76_/BNP_1-32_ after coronary artery bypass grafting surgery.

**Method:** 36 patients were selected purposive (27 men and 9 women with mean of age 60.32 ± 5.81 years, height 164.64 ± 9.25 cm, weight 73.86 ± 14.23 kg, fat 32.30 ± 4.28, SBP 142.67 ± 6.49, DBP 84.5 ± 5.16 mmHg in seated position at rest situation and functional capacity of 7.08 ± 2.49 METs) and then divided randomly into three groups: control (C) group (without training program) moderate continuous training (MCT) and high intensity interval training (HIIT) (exercise training program was performed 3 days/week for 8 weeks) with intensities 65%–80% and 80%–95% of reserve heart rate in order. Blood samples were taken 48 h before the first session and 48 h after the last training session to measure the plasma levels of ProBNP_1–108_, corin enzyme, BNP_1-32_, and NT-pro-BNP_1-76_ using the enzyme-linked immunosorbent assay (ELISA) technique. Wilcoxin and kruskal wallis tests were used for analyzing data.

**Results:** The plasma corin enzyme was increased, and the ratios of proBNP_1-108_/BNP_1-32_ and NT-pro-BNP_1-76_/BNP_1-32_ were reduced in both training groups in compared with control group (*p* = 0.004, *p* = 0000, *p* = 0.016, *p* = 0.003, *p* = 0.009, and *p* = 0.016) when there was no significant difference was found between training groups (*p* = 0.074, *p* = 450, and *p* = 0.295).

**Conclusion:** Both high intensity interval training and moderate continuous training in compared with inactivity have positive effects on ratios of ProBNP_1-108_/BNP_1-32_, NT-pro-BNP_1-76_/BNP_1-32_ and could be effective to promote the health of coronary arteries and prevention of HF in post-CABG patients.

## Introduction

**FIGURE 1:**
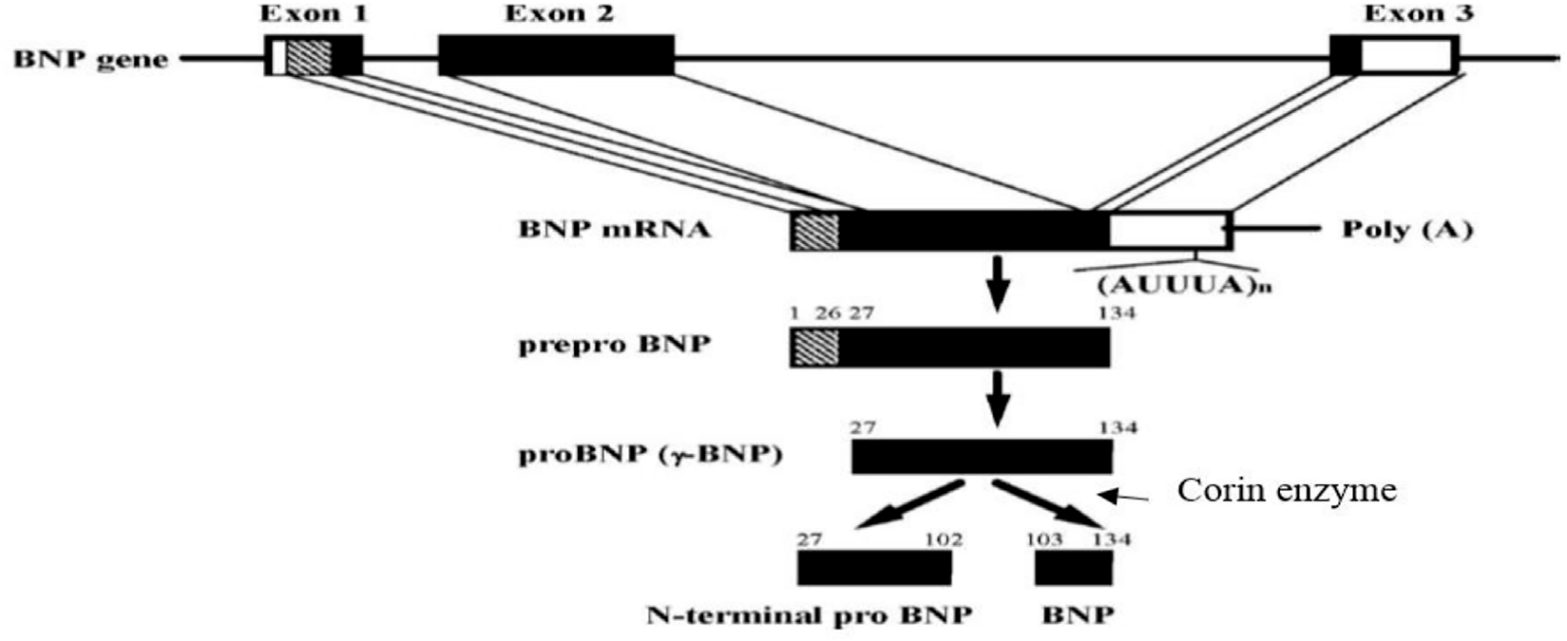
Chemical structure and production processing of BNP_1-32_ and NT-pro-BNP_1-76_.

Coronary artery bypass grafting surgery (CABG) is an important therapeutic strategy for patients with coronary heart disease (Serruys et al., 2001). Although CABG can decrease the rates of morbidity and mortality in patients, by the improvement of blood circulation, in the heart, it is also accompanied by the emergence of some side effects. Postoperative heart failure (HF) is one of the significant outcomes that usually occurs after CABG (Siribaddana, 2012). Several lines of evidence indicate that the complications of CABG include inflammation and the production of free radicals due to reperfusion of the ischemic heart. These factors are the initiator of the fibrosis signaling pathway and negative remodeling of the heart. So, they can weaken the myocardial contractility and cause HF in the long term (Siribaddana, 2012; Cockburn et al., 2013). Brain natriuretic-related peptides (proBNP_1-108_, BNP_1-32_, and NT-pro-BNP_1-76_) are critical markers applied for the diagnosis of HF after surgery (Fox et al., 2011; Preeshagul et al., 2013; Fu et al., 2018a). For example, Fox et al. (2011) showed a direct and strong association between the increase of BNP_1-32_ after CABG and the development of HF in the next 5 years. Fu et al. (2018a) also reported that impairment in processing and degradation of BNP_1-32_ and NT-pro-BNP_1-76_ are effective in the occurrence of HF. Brain natriuretic peptide (BNP_1-32_) is a hormone released from the cardiac ventricles in response to ischemia and myocardial wall stress due to volume or pressure overload (Fu et al., 2018b). BNP_1-32_ is primarily synthesized from an inactive prohormone (proBNP_1-108_) that is cleaved into the active hormone (BNP_1-32_) and the inactive N-terminal fragment (NT-pro-BNP_1-76_) by the enzyme named corin. BNP_1-32_ is decomposed into the body after carrying out its biological function (Ichiki et al., 2011; Ichiki et al., 2013). BNP_1-32_ reduces blood pressure directly and indirectly by relaxing vascular smooth muscles, blocking the cardiac sympathetic nervous system, and increasing the diuretic and natriuretic effects. BNP_1-32_ also inhibits the renin-angiotensin-aldosterone system and has anti-proliferative and anti-fibrotic effects on the myocardium (Chopra et al., 2013). Although BNP_1-32_ is a cardiovascular protective peptide, it is measured in patients’ blood as a cardiovascular stress index. Recent studies indicated that in addition to BNP_1-32_ indices, namely, proBNP_1-108_, NT-pro-BNP_1-76_, like the ratios of ProBNP_1-108_/BNP_1-32_ and NT-pro-BNP_1-76_/BNP_1-32_ play a major role in predicting the development of HF (Jensen et al., 2010; Jensen et al., 2012; Miller et al., 2012; Barnet et al., 2015; Huntley et al., 2015; Fu et al., 2018a). The generation and degradation of BNP_1-32_ is a significant problem in cardiac patients. For example, Barnet et al. (2015) have shown that the amount of corin enzyme involved in the conversion of proBNP_1-108_ to BNP_1-32_ decreases after CABG. Whereas proBNP_1-108_ is increased simultaneously, indicating a gap in the production process of BNP_1-32_, particularly after CABG (Barnet et al., 2015). In other words, the enzymatic activity is impaired in the myocardium of patients, especially after CABG. Hence, the ProBNP_1-108_/BNP_1-32_ ratio could be increased in these patients and may result in an increased risk of HF (Barnet et al., 2015). On the other hand, proper breakdown of BNP_1-32_ and NT-pro-BNP_1-76_ is essential for heart functions; the accumulation of BNP_1-32_ leads to the saturation of its receptor and inefficiency of BNP_1-32_ in patients with cardiovascular diseases (Huntley et al., 2015). Some studies indicated that the impairment of BNP_1-32_ processing and its degradation system could reduce the levels of BNP1-32 and increase the levels of inactive ProBNP1_-108_ and NT-pro-BNP_1-76_. This condition, a paradox of BNP_1-32_, is medically considered a dilemma in patients with cardiovascular diseases (Huntley et al., 2015). Numerous BNP-related peptides are circulated in the bloodstream; however, they have no beneficial effect on the health status of patients with cardiovascular disorders, as most of these peptides are found inactive and have no functionality (Huntley et al., 2015; Zarekarizak et al., 2017). Hence, the ratios of ProBNP_1-108_/BNP_1-32_ and NT-pro-BNP_1-76_/BNP_1-32_ could be regarded as two important indices for BNP_1-32_ processing and degradation, which are defective in patients with cardiovascular diseases. The dysfunction of BNP_1-32_ processing and its degradation retain a fundamental role in the development of HF in the long-term period (Jensen et al., 2010; Jensen et al., 2012; Barnet et al., 2015; Huntley et al., 2015). For example, Suzuki and Sugiyama, (2018) showed a significant risk of HF in patients with a high ratio of NT-proBNP_1-76_/BNP_1-32_ according to Kaplan-Meier analysis (Suzuki and Sugiyama, 2018). In other words, the positive effects of exercise on cardiac rehabilitation, especially ACT, have been addressed and can act as a therapeutic strategy to prevent secondary problems and mortality after surgery (Dendale et al., 2005; Conraads and Beckers, 2010; Valkeinen et al., 2010; Ghashghaei et al., 2012; Meyer et al., 2013; Nishitani et al., 2013). High Intensity Interval Training (HIIT) is also an exercise training employed for cardiovascular adaptations and improvement of functional capacity (Wisloff et al., 2007; Gibala et al., 2012; Guiraud et al., 2012). HIIT is characterized by repeated bouts of high-intensity exercise interspersed by rest periods or low-intensity exercise for recovery (Gibala et al., 2012). HIIT is recommended for patients with coronary heart disease because of the higher tolerability of this type of exercise than continuous exercise programs (Guiraud et al., 2012). In this regard, studies have been performed exercise training on BNP_1-32_ and NT-proBNP_1-76_ levels in cardiac patients. For example, Pearson et al. (2018) reported a positive effect of exercise on BNP_1-32_ and NT-proBNP_1-76_ scores among patients with HF (Pearson et al., 2018). Wisløff et al. (2007) also examined the impacts of eight weeks of high-intensity interval training upon moderate-intensity continuous training. They showed a greater reduction of BNP_1-32_ in high-intensity interval training than in continuous training in HF patients (Wisloff et al., 2007). Additionally, many studies have reported the beneficial impacts of HIIT, but there are conflicts about the optimal training program compared to MCT in patients with coronary heart disease. Besides, the effects of these two training programs remained unclear on the processing and degradation of BNP_1-32_, as shown by the ratios of ProBNP_1-108_/BNP_1-32_ and NT-pro-BNP_1-76_/BNP_1-32_. Therefore, the purpose of this study was to determine the impact of HIIT and MCT on plasma ratios of ProBNP_1-108_/BNP_1-32_ and NT-pro-BNP_1-76_/BNP_1-32_ in patients who underwent CABG. The chemical structure and production processing of BNP1-32 and NT-pro-BNP_1-76_ is depicted in Figure 1.

## Material and methods

### Subjects

The statistical population were all post CABG patients referred to Tehran Heart Center Hospital. Sample size were 36 patients because according to studies there must be at least chosen 30 samples to semi-experimental studies (Dietz and Kalof, 2009). so first of all we selected 36 patients purposive (27 men’s and 9 women) according our criteria and then allocated them to three groups according to homogeneity and gender randomly (9 men and 3 women in each group). The mean of their age were 60.32 ± 5.81 years old, height 164.64 ± 9.25 cm, weight 73.86 ± 14.23 kg, body mass index (BMI) 27.24 ± 3.90 kg/m^2^, systolic blood pressure (SBP) 142.67 ± 6.49, diastolic blood pressure 9 (DBP) 84.5 ± 5.16 mmHg, and functional capacity 7.08 ± 2.49 (METs). Two patients withdrew from the study during the experiment, and 34 patients were remained in our research. Table 3 illustrates patients’ characteristics for each group.

### Inclusion and exclusion criteria

All inclusion and exclusion criteria were considered according to patients’ medical records.

Inclusion criteria were as follows passing one month after following CABG surgery, low cardiovascular risk stratification (Thow, 2006), level 1 hypertension (SBP 140–159 mmHg or DBP 90–99 mmHg or both (Giles et al., 2005), and simultaneous consumption of aspirin, beta-blockers, anti-hypertensive drugs, and statins. Since all subjects were selected with low cardiovascular risk stratification and level 1 hypertension, the dosage of the drugs used by patients was almost the same. Furthermore, all patients had concentric pathologic hypertrophy (LVMI^1^ more than116 g/m^2^ and RWT^2^ was more than 0.42) (Lang et al., 2006).

Exclusion criteria were myocardial infarction, heart valve surgery history, ejection fraction <30%, and movement limitation.

### Procedures

All procedures in this study have been endorsed by the Ethics Committee of Shahid Rajaee Teacher Training University of Tehran (IRSRTTU.SSF.2020.104). Informed consent was obtained from all participants. All measurements were performed as a pre-test after one week of familiarization and 48 h before the first exercise training session. After 2 months and 48 h after the last session of exercise training, all measurements were conducted as a post-test in similar conditions in which the pre-test was carried out. Exercise training was performed along with other routine rehabilitation programs such as psychological rehabilitation (counseling sessions to decrease anxiety and depression of patients), pharmacological treatments (aspirin, beta-blockers, anti-hypertensive drugs, and statins); lifestyle correction counseling (encouragement to increase the physical activity in daily life); diet modification and cessation of cigarette smoking performed in the cardiac rehabilitation clinic.

### Exercise training protocol

**FIGURE 2:**
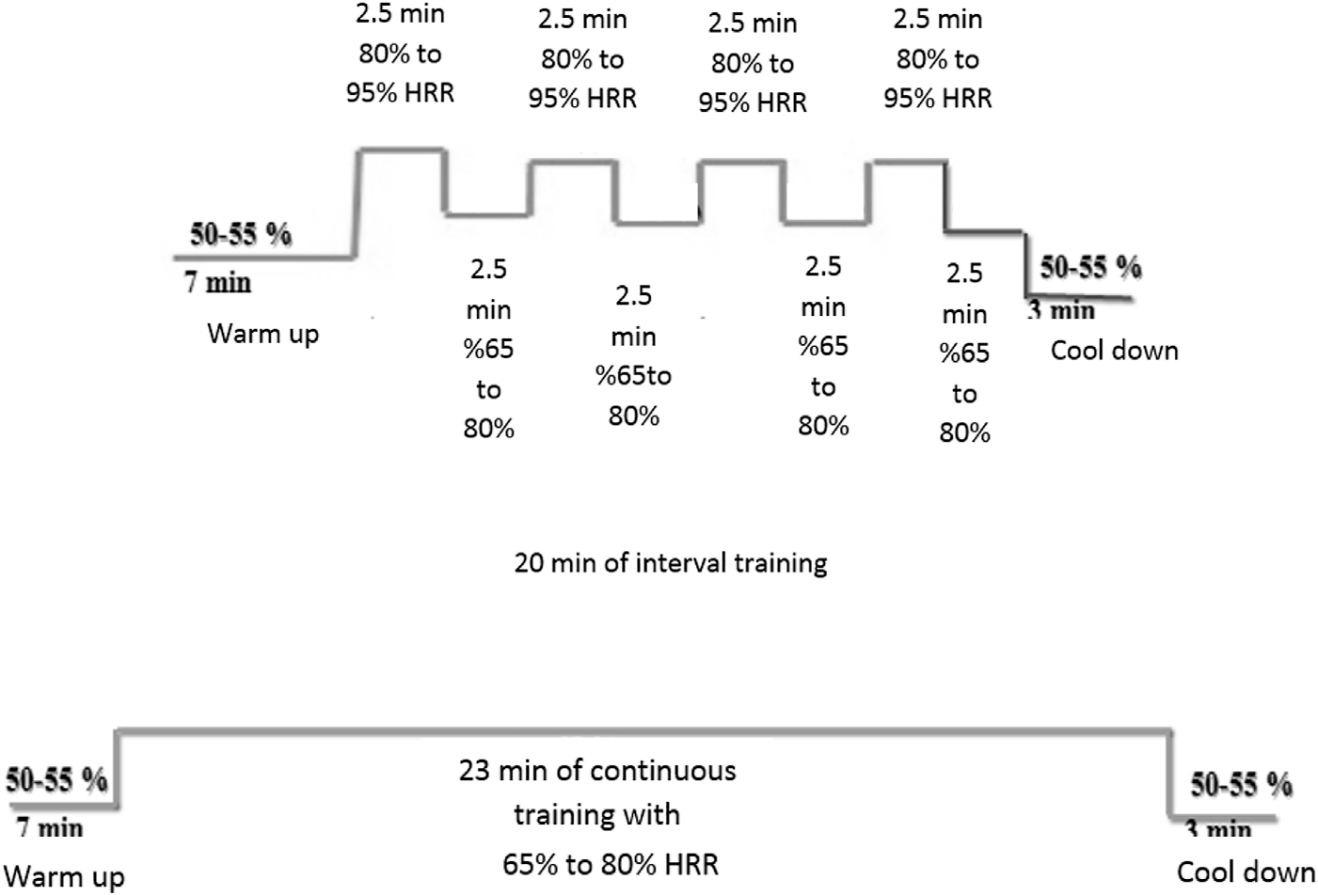
High Intensity Interval Training (HIIT) and Moderate Continuous Training (MCT) protocols.

A comprehensive cardiac rehabilitation program (CR) includes six main aspects: 1) initial patient assessment, 2) nutritional counseling and weight management, 3) ongoing management of coronary risk factors, 4) psychological management, 5) physical activity counseling, and 6) exercise training (de Waard et al., 2021). In the cardiac rehabilitation clinic, exercise training programs were conducted three days a week over 8 weeks training groups performed exercise training on the treadmill (HP Cosmos, Germany) under the supervision of a physician, nurses, and the exercise physiologist. The rate of perceived exertion, rhythm, and arrhythmia was measured by Borg scale, ECG (United States; MHC 1,200), and remote control system (Iran, Avicenna Company; Telemetry), respectively, during each exercise training session. HIIT included 30 min exercises that consisted of 7 min warm-up (walking at 50%–55% of heart rate reserve (HRR), 4 intense intervals for 2.30 min at 80%–95% of HRR, 4 light intervals for 2.30 min at 65%–80% HRR between intense intervals, and finally 3 min cool-down (walking at 50%–55% of HRR). MCT included 33 min exercise on a treadmill that consisted of 7 min warm-up (walking at 50%–55% of HRR), followed by 23 min exercise on a treadmill at 65%–80% HRR, and, finally, 3 min cool-down (walking at 50%–55% of HRR). These protocols were similar to those used in a study by Wisloff et al. (2007) with slight modifications. We considered a similar training load (volume × intensity) to redesign the protocols for HIIT and MCT groups (Figure 2). Furthermore, the mode of intensity overload in the continuous and interval training group have been shown in Tables 1, 2. The maximum heart rate was defined according to the modified Bruce test result. The training heart rate was calculated by the Karvonen equation, as mentioned below (Sagiv, 2012).

HRR=MHR attained−RHR

$$THR=\left(HRR\;*\;\%intensity\right)+RHR$$

HRR, heart rate reserve; MHR, maximum heart rate attained at a peak stress test; and RHR, resting heart rate; THR, training heart rate.

**TABLE 1:** The mode of intensity overload in the MCT group. (HRR: heart rate reserve).

Intensity	Stage	Weeks
60% HRR	Acquaintance stage	0
65% HRR	Initial intensity	1
65% HRR	maintenance	2
70% HRR	Overload	3
70% HRR	maintenance	4
75% HRR	Overload	5
75% HRR	maintenance	6
80% HRR	Overload	7
80% HRR	Maintenance	8

**TABLE 2:** The mode of intensity overload in the HIIT group. (HRR: heart rate reserve).

Moderate intervals	High-intensity intervals	Stage	weeks
60%HRR	75%HRR	Acquaintance stage	0
65%HRR	80%HRR	Initial intensity	1
65%HRR	80%HRR	Maintenance	2
70%HRR	85%HRR	Overload	3
70%HRR	85%HRR	Maintenance	4
75%HRR	90%HRR	Overload	5
75%HRR	90%HRR	Maintenance	6
80%HRR	95%HRR	Overload	7
80%HRR	95%HRR	Maintenance	8

### Laboratory measurements

All participants’ exact height (cm) and weight (kg) were measured for their BMI. Lange Skinfold caliper, 3-site formula, and Siri equation were used to estimate body fat percentage. Resting blood pressure was also measured by the digital blood pressure system (medical space labs made in the United States) from the brachial artery at a seating position. Blood samples (10 mL) were collected into the standard EDTA-containing tubes and immediately transferred to the laboratory to measure values at baseline and after the experiment. The sample plasma was separated by centrifugation at 3,000 rpm for about 10 min, then stored at −80°C until analysis. Corin, BNP_1-32_, proBNP_1-108_, and NT-pro-BNP_1-76_ were measured using the ELISA technique (kits: BOSTER Company made in United States Cat. No: EK1283, BIOMEDICA Company made in Austria Cat. No: BI-20852W and EASTBIOPHARM Company made in china Cat. No: CK-E90422, CK-E10219).

### Statistical analysis

The data were presented as mean and standard deviation (mean ± SD). The Kolmogorov-Simonov test was used to specify the normality of data distribution. We assessed normal distributed data (witness variables: blood pressure and subcutaneous fat, the ratios of proBNP_1-108_/BNP_1-32_ and NT-pro-BNP_1-76_/BNP_1-32_) with the paired sample test and one-way ANOVA to determine within-group variations and between-group differences in the delta of means from pre-test to post-test, respectively. Kruskal-Wallis and Mann-Whitney U tests were used for non-normal distributed data (proBNP_1-108_, Corin, BNP_1-32_, and NT-pro-BNP_1-76_) to analyze within-group variations and between-group differences in the delta of means, from pre-test to post-test, respectively. A *p*-value of less than 0.05 was statistically considered significant for tests.

## Results

The plasma corin enzyme was increased, and the ratios of proBNP_1-108_/BNP_1-32_ and NT-pro-BNP_1-76_/BNP_1-32_ were reduced in both training groups in compared with control group (*p* = 0.004, *p* = 0000, *p* = 0.016, *p* = 0.003, *p* = 0.009, and *p* = 0.016) when there was no significant difference was found between training groups (*p* = 0.074, *p* = 450, and *p* = 0.295).

**FIGURE 3:**
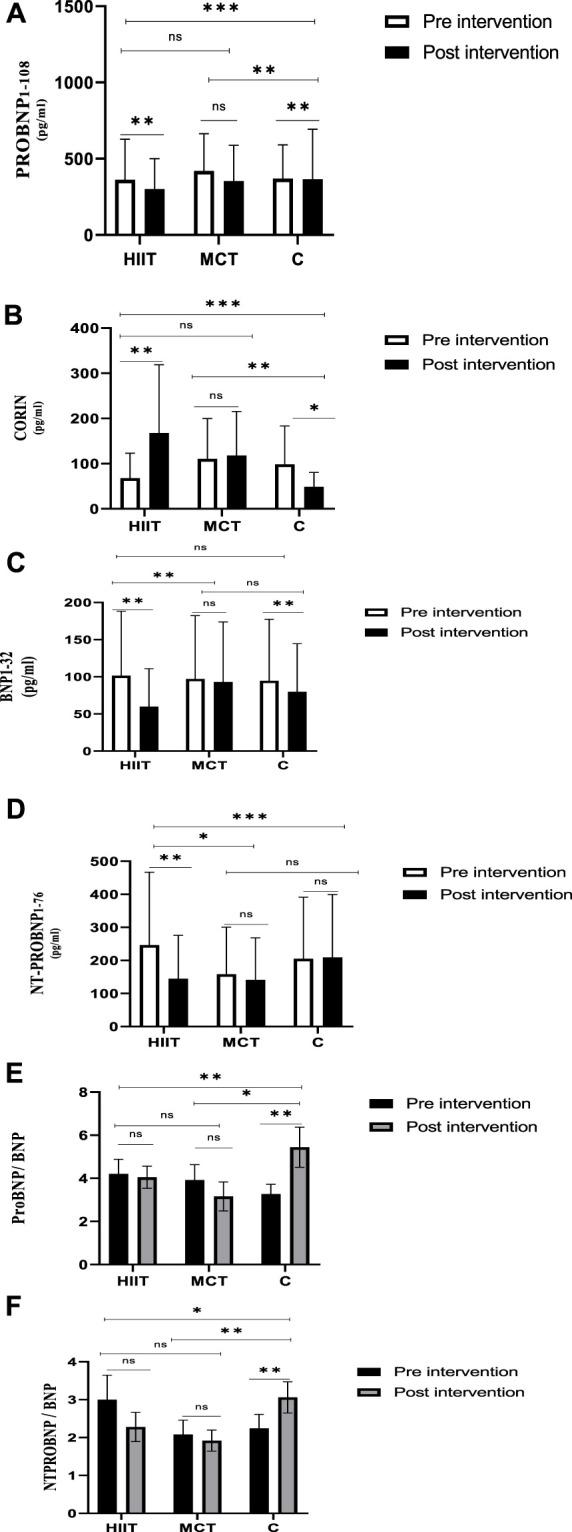
The differences in variables from Pre-Intervention to Post-intervention and between-group differences of variables **(A)** Within and between groups, changes in PROBNP_1-108_. **(B)** Within and between groups, changes in CORIN. **(C)** Within and between groups, changes in BNP_1-32_, **(D)** Within and between groups, changes in NT-PROBNP_1-76_. **(E)** Within and between groups, changes in ProBNP/BNP ratio. **(F)** Within and between groups, changes in NTProBNP/BNP ratio.

The statistical results are depicted in Tables 3–5. Within and between groups, changes in PROBNP_1-108_, CORIN, BNP_1-32,_ NT-PROBNP_1-76,_ ProBNP/BNP ratio and NTProBNP/BNP ratio are shown in Figures 3A–F.

**TABLE 3:** The characteristics of patients.

Variable	Group			*p*-value
	C	MCT	HIIT	
Age (yrs)	60.63 ± 5.58	61.72 ± 4.71	58.75 ± 6.74	0.465
Height (cm)	166.40 ± 12.03	165.81 ± 7.52	161.95 ± 7.82	0.465
Weight (kg)	70.54 ± 13.01	81.54 ± 14.13	69.87 ± 13.60	0.09
BMI(kg/m^2^)	25.95 ± 3.48	29 ± 3.95	26.50 ± 3.86	0.51
Subcutaneous Fat (%)	31.91 ± 3.52	33.34 ± 5.31	31.69 ± 4.0	0.626
Functional capacity (MET)	7.68 ± 2.60	7.02 ± 2.32	7.51 ± 2.45	0.234
SBP (mmHg)	141.18 ± 8.41	143.27 ± 6.23	143.5 ± 4.85	0.661
DBP (mmHg)	83.18 ± 6.32	85.90 ± 4.18	84.41 ± 4.90	0.477

The characteristics of patients were presented as mean ± SD., Differences in characteristics of patients across groups were tested by one-way ANOVA test. HIIT, High intensity interval training group; MCT, Moderate continues training group; C, control group.

**TABLE 4:** Within and between-group differences, analyzed by Wilcoxon, Kruskal-Wallis, and Mann-Whitney *U* tests.

Variable/group	C		MCT		HIIT		Within and between –group			Differences		
	Pre intervention	Post intervention	Pre intervention	Post intervention	Pre intervention	Post intervention	C	MCT	HIIT	*p*-Value		*p*-Value
PROBNP_1-108_ (pg/mL)	774.90 ± 321.28	1,020.30 ± 641.82	858.70 ± 389	801.30 ± 248.83	884.60 ± 189.78	684.10 ± 206.11	0.005*	0.475	0.005*	0.000*	C-MCT	0.003*
											C-HIIT	0.000*
											ACT-AIT	0.034
CORIN (pg/mL)	268.66 ± 156.61	111.55 ± 24.12	288.80 ± 316.95	312.54 ± 309.19	178.50 ± 134.05	470.08 ± 399.94	0.012*	0.131	0.002*	0.000*	C-MCT	0.004*
											C-HIIT	0.000*
											MCT-HIIT	0.074
BNP_1-32_ (pg/mL)	260.30 ± 121.23	209.39 ± 85.18	267.39 ± 129.87	254.77 ± 127.83	274.99 ± 170.16	161.91 ± 52.90	0.003 *	0.091	0.002*	0.022*	C-MCT	0.158
											C-HIIT	0.109
											MCT-HIIT	0.010*
NT-PROBNP_1-76_ (pg/mL)	577.81 ± 274.25	588.90 ± 282.17	443.09 ± 201.04	396.18 ± 155.87	687.16 ± 407.82	407.08 ± 142.83	0.286	0.155	0.002*	0.000*	C-MCT	0.061
											C-HIIT	0.000*
											MCT-HIIT	0.013*
PROBNP_1-108_ BNP_1-32_	3.27 ± 1.51	5.44 ± 3.09	3.92 ± 2.37	3.16 ± 2.23	4.20 ± 2.36	4.05 ± 1.77	0.005*	0.445	0.646	0.006*	C-MCT	0.016*
											C-HIIT	0.003*
											MCT-HIIT	450
NT-PROBNP_1-76_ BNP_1-32_	2.42 ± 1.23	3.06 ± 1.36	2.08 ± 1.26	1.92 ± 0.924	3 ± 2.24	2.38 ± 1.33	0.004 *	0.722	0.099	0.013*	C-MCT	0.009 *
											C-HIIT	0.016*
											MCT-HIIT	0.295
SBP (mmgH)	141.18 ± 8.41	138.54 ± 5.78	143.27 ± 6.23	141.27 ± 6.21	143.5 ± 4.85	124.58 ± 8.46	0.168	0.067	0.000*	0.000*	C-MCT	0.957
											C-HIIT	0.000*
											MCT-HIIT	0.000*
DBP (mmgH)	83.18 ± 6.32	79.63	85.90 ± 4.18	82.81 ± 4.81	84.41 ± 4.90	76.41 ± 4.64	0.086	0.067	0.000*	0.048*	C-MCT	0.978
											C-HIIT	0.125
											MCT-HIIT	0.083
**S**ubcutaneous Fat (%)	31.91 ± 3.52	32.88 ± 4.78	33.34 ± 5.31	32.52 ± 4.29	31.69 ± 4.07	28.90 ± 3.94	0.183	0.239	0.002*	0.002*	CMCT	0.070
											C-HIIT	0.001*
											MCT-HIIT	0.040*

Variables were presented as mean ± SD., The Wilcoxon test tested within-group differences, and differences in variables across groups were tested by Kruskal Wallis and Mann–Whitney U tests. In addition, within-group differences and witness variables (blood pressure and subcutaneous fat) across groups were examined by the paired-sample *t*-test and one-way ANOVA, test. C: control group, HIIT: high intensity interval training group, MCT: Moderate Continues Training group. *: The value of (*p* < 0.05) was considered statistically significant for tests.

**TABLE 5:** Median and IQR of Pre-intervention and Post-intervention of variables in groups.

Group	C				MCT				HIIT			
Variables	Median & IQR of pre intervention		Median & IQR of post intervention		Median & IQR of pre intervention		Median & IQR of post intervention		Median & IQR of pre intervention		Median & IQR of post intervention	
ProBNP_1-108_	675	171	813	183	698.5	254	721	202	830	226	637	232
Corin	240	332	108	24	140	370	200	216	139	228	385	414
BNP_1-32_	227	55	200	50	213.7	184	200.8	246.6	237.9	81	155.1	77
NT-pro BNP_1-76_	462	218	506	297	357	144	339	123	575	520	342	225

Median and IQR of Pre-intervention and Post-intervention of no normal distributed variables in C, control group, HIIT, High-Intensity Interval Training group, MCT, moderate continues training group.

## Discussion

The results of the present study indicated that 8-week HITT, in comparison with MCT, significantly decreased the plasma levels of ProBNP_1-108,_ BNP_1-32_, and NT-pro-BNP_1-76_, on the other hand, increased plasma corin enzyme. Our findings mirror those of similar studies. For instance, Smart and Still (2010) showed the role of resistance and aerobic exercise in reducing BNP_1-32_ and NT-pro-BNP_1-76_ in HF patients (Smart and Steele, 2010). Santoso et al., 2020 have also demonstrated that aerobic exercise can reduce NT-pro-BNP_1-76_ and increase the left ventricular function in HF patients (Santoso et al., 2020). With this line, some studies have shown no effect of exercise on BNP_1-32_ dependent peptides, such as NT-pro-BNP_1-76;_ for example, Zdrenghea et al. (2014) have investigated the effect of two methods, including exercise on the Ergometer and isometric handgrip test on NT-pro-BNP_1-76_ level of HF patients and reported any change. Perhaps the reason for the inconsistency of the mentioned study with the present study was the difference in the kind of patients HF patients *versus* post-CABG patients (in fact, HF is a secondary adverse consequence of CABG in long term. Although HF is a multifactorial problem, it seems hypertension, due to the pressure and volume overload on the heart, and with the direct effect on the structure and function of cardiomyocytes and fibroblasts, causes the gradual development of the fibrotic process, and pathologic hypertrophy. Finally, these structural changes lead to a rise in diastolic heart failure (impairment in optimal ventricular filling), systolic heart failure (impairment in Ejection fraction), and possibly patient mortality (Zile et al., 2011; Zare Karizak et al., 2017). So the improvement of the BNP processing system is important as an effective factor in blood pressure and consequently pathologic hypertrophy and Hf.), type, and duration of exercise (Isotonic and isometric exercise in single sessions *versus* eight weeks of aerobic interval and continuous training). As previously mentioned, BNP_1-32_ is primarily synthesized by the converting enzyme (corin) from an inactive prohormone (proBNP_1-108_) that is cleaved into the active hormone (BNP_1-32_) and inactive N-terminal fragment (NT-pro-BNP_1-76_). So in the present study probably, the reduction of prohormone (proBNP_1-108_) and increase of corin enzyme indicates an improvement in the production system of BNP_1-32_ due to an increase of converting enzyme (corin) in training groups. These results indicated an enhancement in the production system of BNP_1-32_; the amount of BNP_1-32_ and NT-pro-BNP_1-76_, as the final products, decreased in both training groups, especially in the HIIT group. This event may be owing to the regulatory role of BNP_1-32_, which could be increased as a compensatory mechanism in response to pressure overload, volume overload, and ischemic conditions (Fu et al., 2018b). Hence, its concentration is initially elevated in patients with cardiovascular disorders, while it could be decreased after 8-week HIIT. This phenomenon stems from the reduction of stress conditions. Consequently, studies indicated that body adaptation with HIIT could diminish the pressure overload by decreasing vasoconstriction factors, such as angiotensin II, endothelin, and increment in vasodilator agents, such as nitric oxide and prostaglandins (Passino et al., 2006) and decrease of adrenomedullin as a stress index on heart (Zarekarizak et al., 2021). HIIT also decreases volume overload by inhibiting the renin-angiotensin-aldosterone system (Gademan et al., 2007). Other studies have also indicated that HIIT can attenuate the ischemic condition by incrementing the quantity and quality of micro-vessels and improving oxygen delivery to the heart (Macheret et al., 2011). Besides, our findings confirmed that HIIT could dramatically decrease blood pressure compared to other groups. Furthermore, while body fat reduction is associated with an increased ratio of the biological natriuretic peptide receptors (NPR-A to NPR-C), it has been suggested that the body fat percentage influences the sensitivity of the human body to BNP_1-32_ (Dessì-Fulgheri et al., 1997; Marney et al., 2014; Arora et al., 2015). The reduction in body fat percentage after exercise training appears to correlate with the reduction of BNP_1-32_ and an increase in the body’s sensitivity to BNP_1-32_. Our results showed that the rate of the reduction of the body fat percentage is significantly higher at HIIT in comparison with MCT. On the other hand, the ratio of ProBNP_1-108_/BNP_1-32_ was increased in the control group compared with other groups. As previously mentioned, the ratio of ProBNP_1-108_/BNP_1-32_ is an indicator of the production system of BNP_1-32_. The increment ratio in the control group is associated with the impaired production system of BNP_1-32_, which may result from the reduction of the corin enzyme of the control group in the present study. Furthermore, the reduction of degradation enzymes of proBNP_1-108_ has disrupted the BNP_1-32_ production system in the control group likely and increased the ProBNP_1-108_/BNP_1-32_ ratio in this group. In contrast, the ratio of ProBNP_1-108_/BNP_1-32_ was remarkably decreased in plasma levels of both training groups compared with the control group. However, since ProBNP_1-108_/BNP_1-32_ decreased in both training groups, the rate of change in the ratio ProBNP_1-108_/BNP_1-32_ would remain unchanged. Therefore, no significant difference was found in that ratio between training groups. The statistical analysis demonstrated a significant difference in the ratio of ProBNP_1-108_/BNP_1-32_ between the HIIT and control groups and between the MCT and control groups. The ratio of NT-pro-BNP_1-76_/BNP_1-32_ was also increased in the group of control but decreased in the training groups. The ratio of NT-pro-BNP_1-76_/BNP_1-32_ is an indicator of the degradation system of BNP_1-32_. Like the production system of BNP_1-32_, its degradation system was impaired in patients and exacerbated, resulting from the inactivity of the control group. BNP_1-32_ is cleaved in several ways, such as guanylyl cyclase receptor type C and glomerular filtration (Barnet et al., 2015; Huntley et al., 2015), while the breakdown of NT-pro-BNP_1-76_ is exclusively performed by glomerular filtration (Holm, 2013). Also, inflammation is associated with a decreased breakdown of NT-pro-BNP_1-76_ compared to BNP_1-32_ and an increased ratio of NT-pro-BNP_1-76_/BNP_1-32_ (Jensen et al., 2010). Several lines of evidence indicated that exercise training decreases inflammation in post-CABG patients (Goto, 2010), so it could be an influential factor in the degradation of BNP_1-32_ and NT-pro-BNP_1-76_. However, this possible mechanism was not examined in our study and was considered a limitation of the experiment. There was no significant difference between the training groups between ProBNP_1-108_/BNP_1-32_ and NT-pro-BNP_1-76_/BNP_1-32_ ratios. Therefore, both HIIT and MCT may be useful to regulate production and degradation systems for BNP_1-32_ in underwent CABG patients. However, due to the multiplicity of cardiac protection pathways and HF prevention, the lack of more mechanisms was the limitation of this study. For example, BNP1-32 is cleaved by several enzymes, including dipeptidyl peptidase-4 (DPPIV), neprilysin (NEP), and insulin-degrading enzyme (IDE) so further studies are required to survey the effects of MCT and HIIT on these mechanisms. Furthermore, the number of patients was small and this limits the reliability of the results and was limitation of our study. it should be consider more patients for future studies.

## Conclusion

The inactivity of post-CABG patients harms the corin enzyme; ProBNP_1-108_/BNP_1-32_ ratios and NT-pro-BNP_1-76_/BNP_1-32,_ while both HIIT and MCT have a positive effect on ratios of ProBNP_1-108_/BNP_1-32_ and NT-pro-BNP_1-76_/BNP_1-32_ and could be effective to promote the health of coronary arteries and prevention of HF in post-CABG patients.

## Data Availability

The original contributions presented in the study are included in the article/supplementary materials, further inquiries can be directed to the corresponding author.

## Footnotes

1 Left ventricular mass index (LVMI).

2 Relative wall thickness (RWT).
